# Theory of Mind: Overcoming the Dichotomy Between Culture and Nature

**DOI:** 10.5964/ejop.v11i4.1054

**Published:** 2015-11-27

**Authors:** Ruggero Andrisano Ruggieri

**Affiliations:** a Department of Human, Philosophical and Educational Science, University of Salerno, Fisciano, Italy

[Other p1]Confining this book to clinical psychology alone would be a grave mistake for both researchers and practitioners in psychology. In this book, Music (2011), addressing the issue of child development, paves the way towards a theory of the mind that integrates the different psychological approaches and schools.

This integration takes shape through the study of: a) general psychology related to cognitive functions (e.g. memory, language); b) clinical psychology related to the psychological laws as well as normal and pathological conditions; c) developmental psychology related to theories of attachment (Bowlby, 1988, 1969/1999) in relation to both mother and peers (Gorrese & Ruggieri, 2013); d) neuroscience research focused on mirror neurons (Iacoboni, 2009); e) social psychology and, last but not least, f) cultural psychology related to a systemic perspective where the context of relations plays a key role in the understanding of the laws of the functioning of the mind through meaning systems (Ruggieri & Rochira, 2013; Valsiner, 2012, 2013) based on experience (Rosa & Pievi, 2013). Experience becomes the primary means through which nature and culture meet, where biological potential interacts with cultural dimensions, creating different relationship patterns capable of determining the functioning of the mind: in other words, mind and relational patterns are culturally defined (Ruggieri, Pozzi, & Ripamonti, 2014). It is here that the complexity of the author’s thought is realised, overcoming the barriers of individual psychology and the traditional division between Mind/Brain, Individual/Context and Culture/Nature.

Pursuing an understanding of how the mind works, it is necessary to start by understanding its development during childhood. This is the leitmotif of the text that introduces a theory of mind based on systems of meaning that are already present in neo-foetal experience during pregnancy.

There are numerous studies that support this theory, where the inter-subjective dimension of the mind is explored outside the Mind/Brain dichotomy and, for every biochemical process present in the changing body of the mother, there is a change in foetal behaviour as well as the relationship patterns established between mother and child.

‘Born to be related to’ or, better yet, ‘designed to be related to’, this could be the synthesis of the ontological position proposed by Music. Studies conducted on twins show how the interactions between the foetuses – now relatable to patterns of interactions, joining hands, stroking, arguments for more space in the placenta – were entirely reproduced, equally and in the same way after birth as well as during their lifetimes (Piontelli, 1992). For example, Piontelli’s research pointed out how a pair of twins after birth has continued to have exchanges of affection, stroking themselves in the same way and part of the body, using a piece of cloth as the membrane that separated the two bodies during the foetal stage.

More than one study supports this thesis, allowing to assume that there is even an active process of meaning during the period of intrauterine life and that it somehow affects the cognitive functions, as suggested by the idea of ​​forms of memory and language or, broadly, modality of communication, prior to birth. It, therefore, highlights a continuity between life before and after birth, now considered somewhat well-founded from the research in clinical psychology as well as the development of applications for smartphones (e.g. I-Mom, Mom-I) that accompany the expectant mother in the interaction with her foetus, with the environment, with the father.

The hypothesis of a relational and interactive competence of the foetus is confirmed in other studies in which the experience of life in the womb determines and directs the behaviour of the infant. In other words, the individual exists, or rather, the mind exists and takes form only and exclusively in a relational schema given the context in which the experience takes shape.

In Music’s model, the development of the mind goes through a series of continuous and active affective exchanges in the relational processes that are biologically rooted in the activity of mirror neurons (Iacoboni, 2009). They are established as a class of neurons that are activated when an individual performs an action or when an individual observes the same action performed by another person. Music considers their collocation in the area of ​​the brain known as Broca’s area not to be purely casual, since this is also the area associated with such functions as language and imitation. According to this perspective, mirror neurons represent a necessary piece in our understanding of interpersonal functioning and the foundations of human inter-subjectivity, encouraging the development of empathy (Iacoboni, 2009), while also providing answers to the fundamental problem of understanding the mind of others (or mindfulness and Mentalization; see Allen, Fonagy, & Bateman, 2008) and the way with which the integration of information occurs in emotional and cognitive representations of the self, that allow for the modulation and regulation of emotional states, effectively promoting and modulating social behaviour.

The experimental data reported in this book argue the neuroanatomical basis of inter-subjectivity and how the dynamics of sense-making about past experience directs future meaning-making through a series neuronal connections that gradually become stable over time, while maintaining their dynamic and flexible nature, and openness to other possible activities of sense-making. The impact of culture, for example, becomes crucial in understanding this stability/change dynamic. Both the longitudinal and cross-cultural studies presented show how the context of the experience expressed in relationship with parents (Ainsworth, 1978), peers (Gorrese & Ruggieri, 2012, 2013), as well as the culture determine of the developmental pathways of the mind, thus the dynamic of sense-making and neuronal connections.

Culture is not opposed to nature; on the contrary, it actively participates in the development of relational schema on the basis of a clear neuroanatomical foundation that is modulated in the context of the relationship with parents, peers as well as at school and in other social context.

The book is divided into five different parts, with a total of twenty chapters. The first part discusses the issues of the origin of emotional and social development of the mind through the observation of the unborn child, then moving onto its first months of life. The second part is a re-reading of attachment theory in light of the discoveries of neuroscience and the progress of cultural psychology. In the third part, the evolving skills are discussed and placed within an inter-subjective dimension where the use of symbols, language and memory are reflected in play as an expression of a process of sense-making where gender and cultural differences produce different attributions of sense. The fourth part explores the role of the caregiver, not relegated only to the maternal function but expanded to that of the father, siblings and peers. Each person encountered in life becomes a possibility to activate some of the dynamics of sense making, rather than others. The role of experience, due to emotional and social skills, is explored in the fifth and final part of the text, where the effects of early experiences are explored in the long-term consequences on the basis of cultural parameters. How such a set of variables contributes to explaining the outcome of the dynamics of sense making in terms of pathology and normality is discussed.

In summary, a new research perspective opens, through the following claim: there is something in nature that it is in nature, or rather it is nature transformed, from its very emergence, and incessantly through cultural processes. The fecundity of thought leads psychological science to the idea of ​​the complementarity of psychology with other sciences as well as all of psychology (general, social, clinical psychology etc.).

This interdisciplinary approach, though indicated, however, still remains largely unexplored and insufficiently developed, leaving ample room for scientific debate. However, for example, there is no model of sense-making capable of crossing cultural variability according to criteria of validity and reliability, i.e., a model that, using the claim of Music, can explain that is when and how nature becomes culture.

## References

[r1] Ainsworth, M. D. S. (1978). *Pattern of attachment: A psychological study of the strange situation*. Hillsdale, NJ: Lawrence Erlbaum.

[r2] Allen, J. G., Fonagy, P., & Bateman, A. W. (2008). *Mentalizing in clinical practice.* Washington, DC: American Psychiatric Association Publishing.

[r3] Bowlby, J. (1988). *A secure base: Clinical applications of attachment theory*. London, United Kingdom: Routledge.

[r4] Bowlby, J. (1999). *Attachment*: *Attachment and loss* (2nd ed., Vol. 1). New York, NY: Basic Books. (Original work published 1969)

[r5] Gorrese A. Ruggieri R. (2012). Peer attachment: A meta-analytic review of gender and age differences and associations with parent attachment. Journal of Youth and Adolescence, 41, 650–672. 10.1007/s10964-012-9759-6 22476726

[r6] Gorrese A. Ruggieri R. (2013). Peer attachment and self-esteem: A meta-analytic review. Personality and Individual Differences, 55, 559–568. 10.1016/j.paid.2013.04.025

[r7] Iacoboni M. (2009). Imitation, empathy and mirror neurons. Annual Review of Psychology, 60, 653–670. 10.1146/annurev.psych.60.110707.163604 18793090

[r8] Music, G. (2011). *Nurturing natures: Attachment and children's emotional, social and brain development*. London, United Kingdom: Psychology Press.

[r9] Piontelli, A. (1992). *From Fetus to child: An observational and psychoanalytic study*. London, United Kingdom: Tavistock.

[r10] Rosa A. Pievi N. (2013). Semiotic methodology for the analysis of the cultural and individual dynamics of social representations: A view from cultural psychology. Papers on Social Representations*,* 22(2), 19.1-19.29.

[r11] Ruggieri R. Pozzi M. Ripamonti S. (2014). Italian family business cultures involved in the generational change. Europe’s Journal of Psychology*,* 10(1), 79-103. doi:105964/ejop.v10i1.625 10.5964/ejop.v10i1.625

[r12] Ruggieri R. Rochira A. (2013). Semiotics and social representation: A figure-and-ground relationship of mutual cultivation. Papers on Social Representations*,* 22(2), 15.1-15.9.

[r13] Valsiner, J. (2012). Introduction of culture psychology: A renewed encounter of inquisitive of mind. In J. Valsiner (Eds.), *The Oxford handbook of culture and psychology* (pp. 3-24). New York, NY: Oxford University Press.

[r14] Valsiner J. (2013). Creating sign hierarchies: Social representation in its dynamic context. Paper on Social Representations*,* 22(2), 16.1-16.32.

